# Food allergy support groups: member’s profile. Similarities and differences with respect to non-members in the Spanish population

**DOI:** 10.1186/2045-7022-5-S3-O9

**Published:** 2015-03-30

**Authors:** Dasha Roa Medellín, Monleon Alonso Julio, Silvia Calderon Rodriguez, Celia Pinto Fernandez, Elena Alonso Lebrero

**Affiliations:** 1 Hospital Materno Infantil Gregorio Marañón, Madrid, Spain; 2 Santa Barbara, Madrid, Spain

## Background

Children’s food allergy (FA) has impact in the whole family. Patients and caregivers’ Food Allergy Support Groups (FASG) offer expert advice to individuals and families who need help in the management of food allergies, and also contribute in clinical research.

## Objectives

To know clinical differences between members of FASG versus non-members and describe their profiles.

## Methods

We conducted a cross-sectional study including 325 families recruited from a Public Pediatric Allergy Department. Children had been diagnosed of FA at least nine months before, to ensure parental disease experience.

A non-validate questionnaire based on clinical experience, literature records and concerns raised by FASG was elaborated. Ethic Committee approved the study. The questionnaire was filled in the allergy office. Data were stored and processed using SPSS 15.0. Variables were expressed as frequencies and associations using Pearson's chi-square test.

## Results

100% questionnaires were completed (n= 325).

Age: Median 4y 2m (1-15).

80.4% had knowledge about FASG.

13.7% were members.

No statistically significant differences existed between both groups in age, severity, frequency of reactions, comorbidity with asthma and atopic dermatitis, reactions handling and general knowledge about FA.

Statistically significant differences were found in: Allergy to more than 2 foods 74.2% vs 49.6%. Foods: Cow’s milk 59% vs 33.8%, Nuts 38.6% vs 24.5%. Parental perception of economic burden 54.5% vs 24.5%, School problems 39.5% vs 25.8%, Trouble in school meals 62.8% vs 41.6%, Family burden 27.3% vs 14.4 %, increasing housekeeping 59.% vs 27.3%, Impact on family life 70.5% vs 28.7%, Impact on social integration 34.1% vs 12%, social limitations 22.5% vs 9%, Parents’ fear perception about accidental exposures 47.5% vs 27.9%, Misunderstanding labeling 40.9% vs 20.7%, Satisfactory life expectancy perception 81.8% vs 93.8%.

## Conclusions

Perceived burden is higher in FAGS members with respect to non-members.

These results could be explained because the number and type of food allergens are different between both groups and should be taken into account in upcoming trials in collaboration with FAGS.

